# The Correlation Patterns of miRNA Expression with Targeted mRNA Transcripts in Glioma Patients with Wild-Type and Mutated Isocitrate Dehydrogenase (IDH) Genotypes

**DOI:** 10.32607/actanaturae.27363

**Published:** 2024

**Authors:** A. A. Bondarev, A. S. Evpak, A. L. Novoselov, A. A. Kudriaeva, A. A. Jr. Belogurov

**Affiliations:** Shemyakin and Ovchinnikov Institute of Bioorganic Chemistry, Russian Academy of Sciences, Moscow, 117997 Russian Federation; Russian University of Medicine, Department of Biological Chemistry, Ministry of Health of the Russian Federation, Moscow, 127473 Russian Federation

**Keywords:** LGG, low-grade gliomas, microRNA, differential expression

## Abstract

Low-grade gliomas are divided into two main genetic phenotypes based on the presence or absence of mutations in the isocitrate dehydrogenase (*IDH*) genes. The mutated IDH phenotype (IDHmut), in contrast to the wild-type phenotype (IDHwt), is characterized by a more positive response to pharmacological intervention and a significantly longer survival time. In this study, we analyzed the differential co-expression of 225,000 microRNA–mRNA pairs at the level of correlations between microRNA levels and their potential mRNA targets. Analysis of the associative relationships of individual representatives of the selected pairs revealed that the level of mRNAs encoded by the *ELN*, *ARL4C*, *C9orf64*, *PLAT*, and *FKBP9 *genes associated with aggressive progression of glioma was increased in the IDHwt group. Meanwhile, the levels of miRNA-182, miRNA-455, and miRNA-891a associated with the negative prognosis in glioma were generally increased in the IDHmut group. Most (16/21) of the detected 21 microRNA–mRNA pairs with significant difference in regulation between IDHwt and IDHmut glioma samples had a weak or moderate positive correlation in IDHmut samples and a negative correlation in IDHwt samples. Therefore, our findings indicate that glioma samples from the IDHmut group with a positive prognosis potentially have a significantly less pronounced ability to microRNA-mediated regulation. We further suggest that such physiological disorders can lead to reduced tumor viability, resulting in an increased ability of the host to resist the spread of a malignant transformation of this genetic phenotype.

## INTRODUCTION

The incidence rate of glioma is ~ 6.6 per 100,000 population; glioblastoma is diagnosed in almost 50% of cases. The data on the incidence of malignant cerebral neoplasms in the Russian Federation are rather inconclusive. According to various estimates, the incidence rate of these malignant neoplasms (MNs) can be as high as 23 cases per 100,000 population; the incidence of glioma is 10–13 cases per 100,000 population [1]. The risk of developing this pathology increases abruptly with age: from 0.15 in childhood to 15 per 100,000 population in the elderly aged 75–84 years [2].

The reasons for the increasing incidence of glioma have yet to be fully elucidated. That is possibly related to the mass-scale introduction of high-tech methods for diagnosing malignant cerebral neoplasms, such as magnetic resonance imaging and positron emission tomography, into clinical practice [2]. Multiple external environmental factors have been considered as a reason for the emergence of glioma; however, the statis tically significant rise in the risk of glioma emergence is now believed to be associated exclusively with ionizing radiation [3, 4, 5].

MicroRNAs are small noncoding RNA molecules that regulate gene expression by binding to mRNA targets, thus causing their degradation or translation inhibition [6]. Numerous studies have revealed significant changes in microRNA expression during malignant transformation. Taking these results into account, microRNAs are currently being offered as potential diagnostic or prognostic biomarkers. Expression of microRNAs in humans with malignant neoplasms is disrupted via different mechanisms such as amplification or deletion of microRNA genes, abnormalities in microRNA transcription regulation, as well as epigenetic changes and defects in the mechanisms of microRNA processing. microRNAs can be classified as oncogenes or tumor suppressor genes.

The genetic features of gliomas are intensely used for tumor classification and selection of the optimal treatment strategy. Several attempts to characterize low-grade gliomas with wild-type and mutated isocitrate dehydrogenase (*IDH*) genes using microRNA signatures have been made [7, 8, 9]. In this study, we investigated the correlation patterns of microRNA coexpression with their potential targeted transcripts in patients with low-grade gliomas with wild-type and mutated *IDH *phenotypes.

## EXPERIMENTAL

**Data Sources**

The TCGA-LGG cohort (https://portal.gdc.cancer.gov/ projects/TCGA-LGG) containing the versatile genome sequencing data of individual patients with LGG, as well as data on gene and miRNA expression, was used as the source data to analyze low-grade gliomas.

**Software**

The analysis was conducted using standard tools for processing transcriptome data for the Python 3.10 programming language. The packages RNAnorm 2.1.0 and PyDESeq2 0.4.4 were used for data normalization and preprocessing. The correlation coefficients were calculated using the package SciPy v1.12.0. The survival curves were plotted using the package Lifelines 0.28.0.

**Block diagram**

**Fig. 1:**
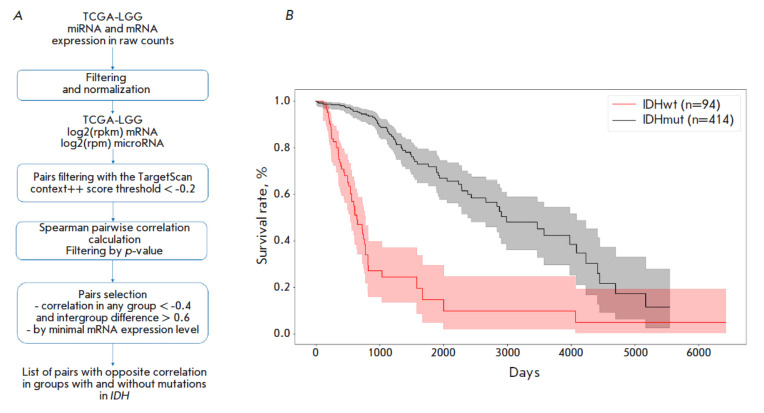
(*A*) Block diagram of the bioinformatic pipeline. (*B*) The survival curve of TCGA-LGG patients divided into two molecular subtypes: IDHwt (a group carrying no mutations in the IDH genes) and IDHmut (a group carrying mutations in the IDH genes)

*Figure 1A* shows the block diagram of the pipeline. Step sequence involves filtering according to the gene expression level, filtering across the TargetScan database with a certain confidence level (context++ score threshold < -0.2), correlation analysis, and pair selection using the correlation coefficient level as a criterion.

## RESULTS

It has been demonstrated earlier that the survival time of LGG patients correlates with the presence/absence of mutations in the *IDH *genes and presence/absence of a deletion in chromosomes 1p and 19q. Based on these data, it has been suggested that LGGs can be subdivided into three molecular subtypes: IDHwt – non-mutated *IDH *genes; IDHmut-no-codel – mutation in the *IDH *genes and absence of deletions in chromosomes 1p and 19q; and IDHmut-codel – mutations in the *IDH *genes and deletions in chromosomes 1p and 19q [10]. In this study, we settled upon two groups: the group with the wild-type *IDH *phenotype and the group carrying mutations in the *IDH *genes (IDHmut). We used data on the survival time of individual patients and previously published data on the molecular subtypes of LGG to analyze patient survival time in the cohorts. The recorded Kaplan–Meier curves agree well with the data published previously and demonstrate that survival time in patients with the wild-type *IDH* genotype was much shorter than that in patients with the mutated *IDH* phenotype (*Fig. 1B*).

The biological role of microRNA has been conventionally studied via differential gene expression analysis by isolating microRNAs characterized by significant intergroup differences in the average expression levels. However, these methods fail to detect changes in the cases when the average expression levels of regulatory and targeted RNAs remain unchanged. Differential co-expression analysis, which detects gene pairs or clusters whose co-expression changes between groups, can be used in this case [11]. These changes can attest to a loss of regulation between microRNA and its mRNA target due to mutations (e.g., in the binding site). Such parameters as parametric Pearson correlation or Spearman’s rank correlation are used to quantify the co-expression level. By comparing these parameters in different groups, one can draw a conclusion that co-expression of a particular pair has been considerably changed. We searched for characteristic microRNAs whose regulatory function significantly changes in the IDHwt and IDHmut groups. Differential co-expression analysis revealed a difference in the regulation of the gene expression level by microRNAs potentially interacting with their transcripts depending on the presence/absence of mutations in the *IDH *genes.

There is intense clinical research under way into LGGs; however, comprehensive analysis of largescale cohorts involving sequencing of tumor DNA and patients’ DNA, as well as analysis of gene expression and DNA methylation, is virtually nonexistent. The only study of TGG samples collected from 530 patients has been deposited into the Cancer Genome Atlas (TCGA) (https://portal.gdc.cancer.gov/). We performed pre-filtering of all the possible pairs for microRNA–mRNA interactions obtained both experimentally and *in silico *across the existing databases. TargetScanHuman 8.0 (https://www.targetscan. org) for finding mRNA targets of microRNAs was used as the main database. At the second stage, we calculated the correlation of expression between protein- coding mRNAs and all the microRNAs. Pairs for which Spearman’s correlation coefficient was < -0.4 in any of the groups (IDHwt or IDHmut), which corresponds to a strong negative correlation (namely, significant effect of this microRNA on gene expression), were considered significant. The absolute difference in intergroup correlation coefficients should be ≥ 0.6.

The result yielded 169 pairs (156 different mRNAs).

**Fig. 2:**
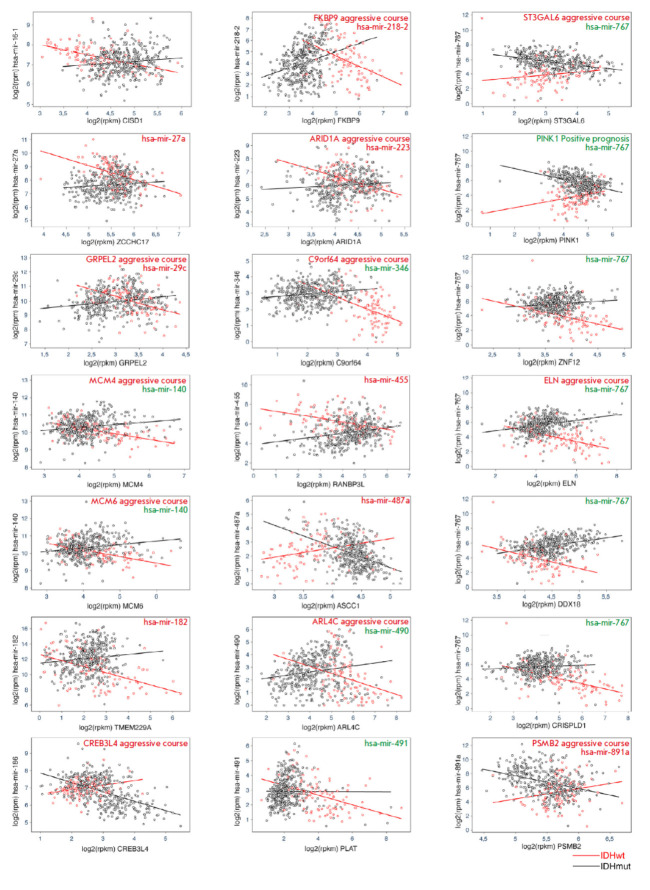
Graphs of microRNA–mRNA correlations for 21 pairs. Black is the IDHmut group; red is the IDHwt group. Expression levels for each patient and linear regression for each group are shown. mRNAs and microRNAs associated with a negative prognosis of MN are marked in red; protective ones, in green. RPM – reads per million mapped reads; RPKM – reads per kilobase per million mapped reads

At the final stage, the pairs were filtered by the significance level of the correlation coefficient in each group (*p * < 0.05), by the mRNA expression level (≥ 2 in the log2 (RPKM) scale), and by the difference in correlation coefficients (≥ 0.6). *Figure 2* shows the data on dependence for 21 microRNA–mRNA target pairs that have passed through all the filtration stages. The expression levels for each microRNA–mRNA target pair in each sample are shown in a color corresponding to the IDHwt and IDHmut groups. For illustrative purposes, we provide linear regression for the IDHwt and IDHmut groups, where one can observe that their regulation patterns are differently directed. Color designation was used for the names of microRNA and mRNA: RNAs associated with the negative prognosis of LGG are shown in red; protective ones are shown in green. The data were obtained from earlier publications and are described in more detail in the Discussion section.

**Fig. 3:**
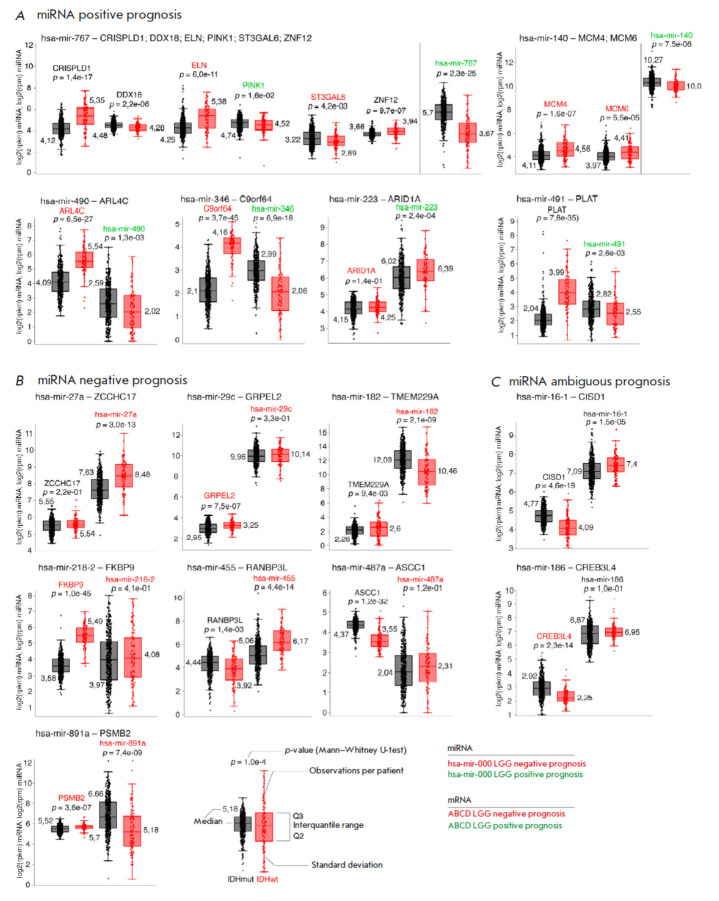
Expression levels for 21 microRNA–mRNA pairs, grouped according to known data on the positive (*A*) and negative (*B*) effects of microRNAs on the disease course. mRNAs and microRNAs associated with a negative prognosis of MN are shown in red; protective ones, in green. The black diagrams are the IDHmut group; the red diagrams are the IDHwt group

*Figure 3* shows the expression levels in these pairs. The microRNA–mRNA pairs are clustered into three groups according to the prognosis of their effects on the disease course (in accordance with the published data); the color designation of names is similar to that used in *Fig. 2*. The expression levels of each pair in the samples collected from each patient, their median value for a group, and the level of significance of differential expression between the IDHwt and IDHmut groups calculated using the Mann–Whitney test are presented. Detailed analysis of the role of expression levels in the pairs was conducted in the Discussion section.

Interestingly, a strong negative correlation between protein-coding mRNAs and miRNAs is observed in the group without mutations in the *IDH *genes and either no correlation or a positive correlation is observed in the group of patients carrying a mutation in the *IDH *genes in the vast majority of cases, which potentially attests to the loss of a functional association between miRNA and mRNA targets.

**Table 1:** A list of miRNA–mRNA pairs filtered by the difference in correlation coefficients

mRNA	miRNA	Correlation (IDHmut)	Correlation (IDHwt)	Difference in correlation coefficients	TargetScan context++ score
DDX18	hsa-mir-767	0.365	-0.432	0.797	-0.325
PINK1	hsa-mir-767	-0.453	0.316	0.769	-0.239
ELN	hsa-mir-767	0.322	-0.442	0.764	-0.222
FKBP9	hsa-mir-218-2	0.319	-0.441	0.76	-0.243
CREB3L4	hsa-mir-186	-0.507	0.247	0.754	-0.25
ASCC1	hsa-mir-487a	-0.402	0.338	0.74	-0.217
ST3GAL6	hsa-mir-767	-0.406	0.308	0.714	-0.247
MCM6	hsa-mir-140	0.199	-0.508	0.707	-0.319
RANBP3L	hsa-mir-455	0.298	-0.405	0.703	-0.361
PSMB2	hsa-mir-891a	-0.405	0.297	0.702	-0.319
MCM4	hsa-mir-140	0.176	-0,512	0.688	-0.311
GRPEL2	hsa-mir-29c	0.208	-0.478	0.686	-0.306
ZNF12	hsa-mir-767	0.144	-0,521	0,665	-0.215
ARL4C	hsa-mir-490	0.237	-0.42	0.657	-0.241
ZCCHC17	hsa-mir-27a	0.111	-0.532	0.643	-0.221
PLAT	hsa-mir-491	0.195	-0.439	0.634	-0.252
CRISPLD1	hsa-mir-767	0.12	-0.51	0.63	-0.314
CISD1	hsa-mir-16-1	0.114	-0.51	0.624	-0.451
ARID1A	hsa-mir-223	0.147	-0.469	0,616	-0.232
C9orf64	hsa-mir-346	0.178	-0.438	0.616	-0.231
TMEM229A	hsa-mir-182	0.129	-0.474	0.603	-0.264

*Table 1* lists the numerical data for the obtained pairs.

## DISCUSSION

MicroRNAs are small, single-stranded noncoding RNAs (20–23 nucleotides long) that are involved in oncogenesis, as well as progression and metastatic spread of various tumors as they regulate a large number of transcripts [12]. microRNA expression is altered in many brain tumors, including both lowgrade gliomas and the most common and malignant subtypes of glioblastoma [13]. microRNAs are associated with the key processes in gliomas such as cell proliferation, apoptosis, and invasion [14].

Mutations in the *IDH *genes are the main genetic marker characterizing the aggressiveness of gliomas. Patients with the wild-type *IDH *phenotype have a negative prognosis, whereas mutations in the *IDH* genes are associated with increased survival time [15, 16, 17].

This study assessed potential dysregulation of the physiological function of miRNA in the IDHwt and IDHmut groups. Most of the 21 detected miRNA– mRNA pairs with differential co-expression (16/21) are characterized by either weak or moderate positive correlation between protein-coding mRNAs and miRNAs in IDHmut samples and by negative correlation in IDHwt samples.

In high-grade gliomas, expression of miRNA-767 (which is considered protective in glioma patients) is significantly lower compared to low-grade gliomas and healthy tissues [18, 19]. According to our data, the miRNA-767 level is reduced in the IDHwt group compared to IDHmut and correlates negatively with the transcripts of such genes as *DDX18 *(RNA helicase),* ELN *(connective tissue protein responsible for elasticity),* ZNF12 *(transcriptional repressor), and *CRISPLD1* (extracellular vesicle protein), while positively correlating with mRNA of *PINK1 *(kinase involved in mitochondrial protein phosphorylation) and *ST3GAL6 *(sialyltransferase) in samples with the wild-type *IDH1* phenotype. Importantly, the upregulated expression of the *ELN *[20] and *ST3GAL6 *genes [21] is associated with the more aggressive type of gliomas and, therefore, lower survival time. Meanwhile, gliomas characterized by downregulated expression of the protective* PINK1 *gene correlate with low survival time of patients who have undergone chemotherapy or radiation therapy [22]. Currently, no data are available on the role played by the *DDX18*, *ZNF12*, and *CRISPLD1* genes in patients with gliomas and low-grade gliomas in particular.

According to the published data, expression of miRNA-218-2 [23], 487a [24], 891a [25], 29c [26], 27a [27], 182 [28], and 455 [29] is elevated in aggressive gliomas or is associated with a negative prognosis, while miRNA-140 [30], 490 [31], 346 [32], and 223 [33] facilitate the inhibition of tumor proliferation. The findings on miRNA-16-1 expression are rather inconclusive: its level is reduced in gliomas with the mutated* IDH1 *phenotype compared to wild-type *IDH1* tissues; meanwhile, reduced expression of this miRNA contributes to tumor proliferation [34, 35]. No credible information about the role of miRNA-186 in patients with gliomas could be found. We observed a statistically significant difference between the studied IDHwt and IDHmut groups in terms of the expression of the aforementioned miRNAs for miRNA-182, miRNA-455, and miRNA-891a, whose levels were significantly reduced in glioma samples in the IDHwt group. Contrariwise, the miRNA-455 level was increased in the IDHwt group compared to IDHmut glioma samples.

Overexpression of the genes whose transcripts act as potential targets for dysregulated miRNAs in the IDHmut and IDHwt groups is observed in more aggressive glioma types: *FKBP9 *[36] – *CREB3L4 *[37],* MCM4*, *MCM6 *[38], *PSMB2 *[39], *ARID1A *[40], *ARL4C* [41], *GRPEL2 *[42], and *C9orf64 *[43]. No data on the involvement of such genes as *ASCC1*, *ZCCHC17 *(participates in biogenesis of ribosomal DNA), *PLAT*, *CISD1*,* TMEM229a *and *RANBP3L *in the development and spread of any glioma types have been found. The conducted analysis revealed significant elevation of the levels of mRNAs encoding the ELN, ARL4C, C9orf64, PLAT, and FKBP9 proteins in IDHwt samples compared to the IDHmut group.

## CONCLUSIONS

Our study has demonstrated that differential co-expression analysis can be successfully used to search for physiologically significant miRNA–mRNA pairs in groups of patients with LGGs and different *IDH *mutational phenotypes. The revealed patterns demonstrate that the mRNA level is elevated in the IDHwt group, which is typical of the aggressive progression of gliomas. Meanwhile, the level of miRNAs associated with a negative prognosis in glioma patients is generally increased in the IDHmut group, which is characterized by much higher chances of survival, once again attesting to the intricate pattern of formation of transcriptional regulatory networks. Nonetheless, at the level of associative relationships, glioma samples from the IDHmut group with a positive prognosis have a much smaller regulation ability. These physiological disruptions may reduce tumor viability and, therefore, improve the ability of a host to resist progression of malignant neoplasms.

## Abbreviations

LGG: low-grade gliomas
MN: malignant neoplasm
IDH: isocitrate dehydrogenase
